# Synergistic associations of visual and self-reported hearing acuity with low handgrip strength in older adults: a population-based cross-sectional study

**DOI:** 10.1186/s12877-021-02470-w

**Published:** 2021-09-25

**Authors:** Seung Hoon Kim, Kyungduk Hurh, Yoonsik Park, Sung-In Jang, Eun-Cheol Park

**Affiliations:** 1 Department of Preventive Medicine, Yonsei University College of Medicine, 50-1 Yonsei-ro, Seodaemun-gu, Seoul, 03722 Republic of Korea; 2 Institute of Health Services Research, Yonsei University, Seoul, Republic of Korea; 3 Department of Public Health, Graduate School, Yonsei University, Seoul, Republic of Korea

**Keywords:** Visual acuity, Hearing acuity, Sensory impairment, Handgrip strength, Sarcopenia, Muscle weakness

## Abstract

**Background:**

It is unclear whether visual and hearing acuity are independently or synergistically associated with muscle strength. We aimed to examine the associations of visual and self-reported hearing acuity with low handgrip strength and the additive interaction between visual and hearing acuity on low handgrip strength in people over 60 years.

**Method:**

Data of 3,075 individuals aged over 60 years from the 2017 and 2018 Korea National Health and Nutrition Examination Survey were used for this cross-sectional study. Low handgrip strength was defined based on the 20th percentile of the study population (< 30.4 kg for male and < 17.7 kg for female). Visual and self-reported hearing acuity were each divided into three categories: good, moderate, and impaired. Multiple logistic regression and relative excess risk due to interaction (RERI) were performed.

**Results:**

Of the 3,075 participants, 993 (32.3 %) demonstrated low handgrip strength. Low handgrip strength was more prevalent in participants with moderate (adjusted odds ratio [AOR] = 1.54, 95 % confidence interval [CI] = 1.12–2.12) and impaired visual acuity (AOR = 2.00, 95 % CI = 1.34–2.96). Both moderate and impaired self-reported hearing acuity were significantly associated with low handgrip strength (moderate: AOR = 1.25, 95 % CI = 1.01–1.55; impaired: AOR = 1.66, 95 % CI = 1.15–2.38). The more severe the sensory function decline, the higher the association with muscle weakness. Moreover, combined sensory impairments were associated with deteriorating low handgrip strength (AOR = 8.38), with significantly strong additive interactions (RERI = 2.61, 95 % CI = 2.52–2.70).

**Conclusions:**

Awareness is needed regarding the risk of reduced muscle strength in individuals with moderate and impaired sensory function. Older people with sensory function decline in clinical settings may benefit from programs such as exercise prescription to prevent muscle weakness.

**Supplementary Information:**

The online version contains supplementary material available at 10.1186/s12877-021-02470-w.

## Background

Age-related decline of physical function and increasing functional disability are associated with adverse health outcomes in older people, including reduced quality of life and increased all-cause mortality [1, 2]. Among the indicators of physical performance, such as gait speed, balance, and chair stand tests, handgrip strength employs simple and noninvasive measures to provide an estimate of the isometric strength of the upper limb [3]. Handgrip strength is considered an estimate of “overall strength” because it is related to the strength of other muscle groups [4]. Moreover, handgrip strength is also a useful predictor of nutritional status and an objective component of the frailty syndrome in older people [5–7]. Low handgrip strength (LHGS) is associated with reduced quality of life [8], and recent studies have shown that older people with LHGS are at a higher risk of suffering from disabilities and are more likely to be hospitalized [9, 10]. Furthermore, handgrip strength is an independent predictor of all-cause mortality and cardiovascular diseases [11, 12].

Visual and hearing impairment increase with age. In the older Korean population (aged ≥ 60 years), the prevalence of hearing loss is 16.8 % [13], and visual impairment is also a common problem [14]. Sensory impairments, including visual and/or hearing impairments, are associated with several problems that can arise in old age. Primarily, research has reported an association between vision and increased frequency of falls and an increase in the risk of recurrent falls in older women due to visual impairment [15, 16]. Furthermore, greater frailty, measured by gait speed, grip strength, peak expiratory flow rate, and the ability to rise from a chair without arm support, is associated with poorer visual functions [17]. Several studies have shown that older people with hearing impairment have increased functional disability, reduced ability to perform activities of daily living (ADL), and increased risk of frailty [18, 19].

Furthermore, the problem becomes aggravated if visual and hearing impairment occur concomitantly. Combined visual and hearing impairment have a greater effect on the functional status of ADL and instrumental activities of daily living than solitary sensory impairment [20]. Additionally, those with combined visual and hearing impairment report higher mortality risk than those with only a single sensory impairment [21].

Despite the functional connection between sensory and motor areas [22], little is known about the relationship between visual and hearing acuity, muscle weakness, and low muscle mass, which is an important cause of the decrease in physical function [4, 23, 24]. Sensory function is an indicator of healthy physical aging. Considering the common-cause theory, which suggests that generalized aging effects can simultaneously contribute to the development of sensory organ dysfunction and reduced muscle strength [25], we proposed that the decrease in visual and hearing acuity is related to LHGS. In addition, if a synergistic effect appears when the impairments of the two sensory organs are added, the priority of intervention will be different from a clinical and public health perspective. Furthermore, unlike handgrip strength measurement, gait speed—a frailty index—particularly requires sight- and hearing-based movement [26]. Therefore, it may be difficult to confirm the decline of physical function through the gait speed of older adults with only sensory impairment, which establishes the need to confirm the relationship between sensory organ function and grip strength.

Moreover, studies evaluating the association between sensory function and handgrip strength divided sensory function dichotomously (mainly into good or impaired); thus, the dose-response effect of sensory function decline on handgrip strength could not be evaluated [23, 24]. Owing to the sex difference in predictors of handgrip strength and association of age, physical activity, and body mass index (BMI) with LHGS in older Koreans [27], it is also necessary to consider these points for examining the relationship between sensory function decline and LHGS.

Therefore, we aimed to evaluate the association of visual and self-reported hearing acuity with LHGS in older Koreans. The current study used a measure that has more than two levels, which allows more sensitivity in comparing various degrees of impairment with those with normal acuity. Furthermore, we investigated the interaction of visual and hearing acuity with LHGS and performed subgroup analyses stratified by sex, age, physical activity level, and BMI.

## Methods

### Study population and data

This cross-sectional study used data obtained from the 2017 and 2018 Korea National Health and Nutrition Examination Survey (KNHANES), which is a nationwide population-based survey designed to acquire information regarding the health and nutrition of people in South Korea [28]. The survey was performed by the Korea Diseases Control and Prevention Agency (KCDA) and combines a health interview with a physical examination and nutrition survey. The trained staff such as doctors or medical technicians conducted the health interview and physical examinations at the mobile examination center, and the dieticians visited the households for nutritional surveys [29]. Complex and multi-stage probability sample design was used in KNHANES to provide an unbiased cross-sectional estimate of Korean population [29]. The detailed description of KNHANES has been published elsewhere [29, 30]. The KNHANES survey protocols were approved by the Institutional Review Board of the KCDA (IRB No. 2018-01-03-P-A), and the research complied with the tenets of the Declaration of Helsinki for medical research involving human participants. Written informed consent was obtained from all participants in KNHANES [31].

**Fig. 1:**
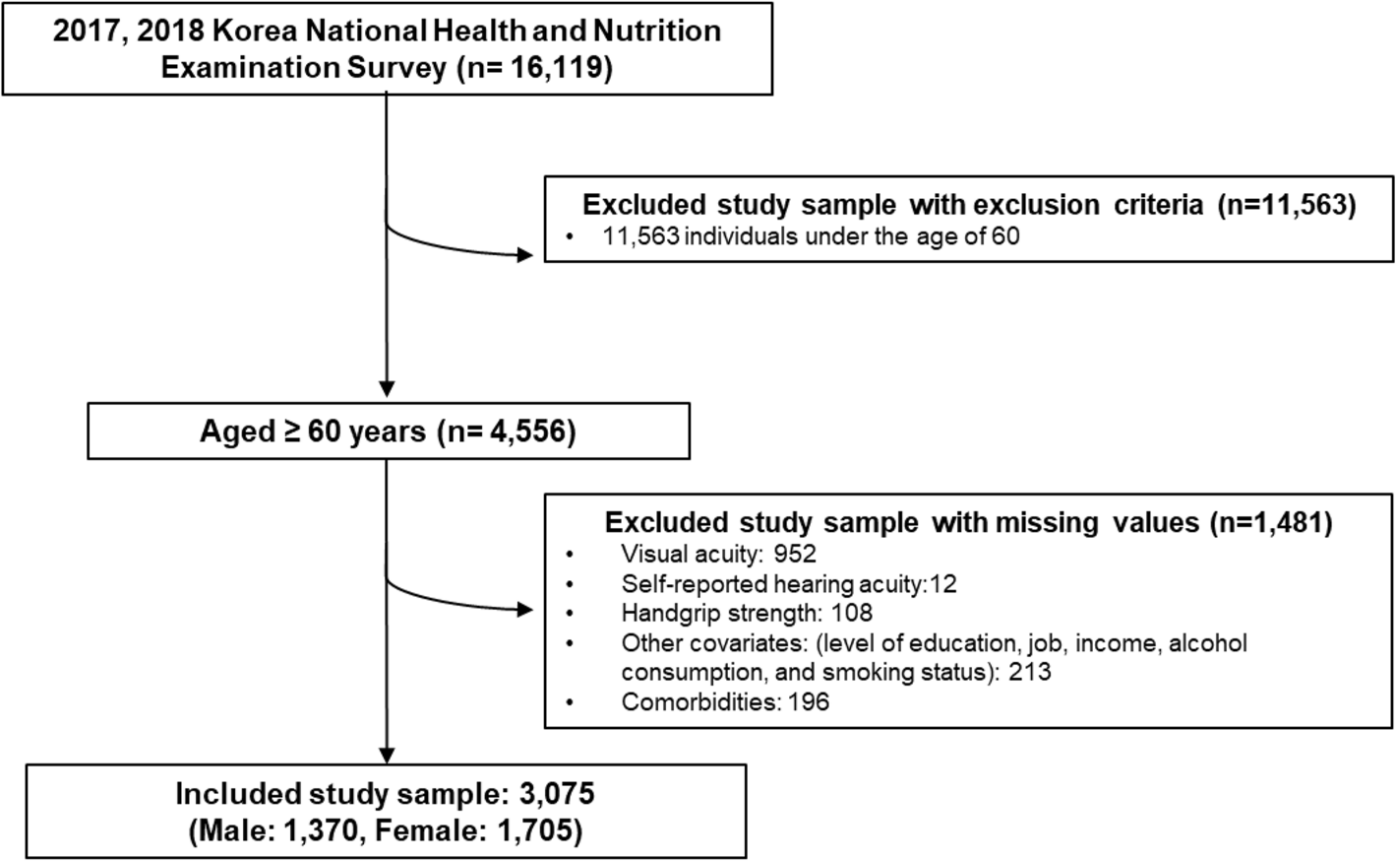
Flow chart for study sample

A total of 16,119 participants were involved in the 2017–2018 KNHANES. In this study, persons aged < 60 years were excluded (n = 11,563) because we aimed to evaluate the association of visual and hearing acuity with LHGS in older Koreans; those who did not undergo visual acuity evaluation or assessment via an auto-refractor/keratometer were also excluded (n = 952). Furthermore, those who answered “do not know,” refused to respond to the questions, or had missing data for the question, “Which statement best describes your hearing (without a hearing aid)?” were excluded (n = 12). Additionally, persons whose handgrip strength data were unavailable and those who did not answer the query regarding the dominant hand were excluded (n = 108). Finally, after excluding participants with missing variables in covariates in this study (n = 409), 3,075 participants (1,370 male and 1,705 female participants) were selected (Fig. 1).

### Variables

The main dependent variable was LHGS. Handgrip strength was measured using a digital grip strength dynamometer (T.K.K 5401; Takei Scientific Instruments Co., Ltd., Tokyo, Japan) and the following method was employed: after determining the dominant hand for each participant through verbal queries, both hands of the participants were measured thrice alternately, starting with the dominant hand, which was followed by a resting period of 60 s. For measuring handgrip strength, participants were instructed to face forward while standing upright, straighten the shoulders, let both arms hang straight down naturally, and grasp the dynamometer with no flexion or extension of the wrist and elbow during the measurement. The maximum handgrip strength of the dominant hand was measured and used for the analysis. The criteria for LHGS were based on the 20th percentile of the study population [32]. Consequently, LHGS was defined as < 30.4 kg for male and < 17.7 kg for female in this study.

The independent variables were visual and self-reported hearing acuity. Visual acuity was measured at a distance of 4 m using an international standard vision chart based on the Snellen scale, called the Jins vision chart [33]. Participants’ visual acuity pertaining to each eye was evaluated, right eye followed by the left, with his or her existing refractive correction (if applicable). In cases where the measured visual acuity was < 0.8 in at least one eye based on the Jins vision chart, the participants underwent automated refraction using an auto-refractor/keratometer (KR8800; Topcon, Tokyo, Japan). Participants’ visual acuity was defined as the best corrected visual acuity (BCVA) based on the eye with the best acuity. Visual acuity was classified into three categories as follows: 0.8 (20/25) ≤ BCVA ≤ 1.0 (20/20) [good], 0.5 (20/40) ≤ BCVA ≤ 0.63 (20/32) [moderate], BCVA < 0.5 (20/40) [impaired] [34, 35]. Hearing acuity was measured as self-reported by the following question: “Which statement best describes your hearing (without a hearing aid)?” The responses were divided into three groups as follows: good (good), moderate (moderate trouble), and impaired (considerable trouble and almost deaf) [36]. Furthermore, owing to the lack of an established three-level classification standard for self-reported hearing acuity, hearing acuity accounting for the use of a hearing aid was defined as follows for the sensitivity analysis: good (good or normal hearing without hearing aid), moderate (moderate trouble without hearing aid), and impaired (moderate or considerable trouble with hearing aid and almost deaf).

Sociodemographic, health-related factors, and year of survey (2017, 2018) were all used as covariates in our study [37]. In sociodemographic factors, age was categorized into four groups as follows: 60–64 years, 65–69 years, 70–74 years, and $\ge$75 years. Other sociodemographic factors were categorized as follows: region (rural and metropolitan), educational level (under high school and college degree or above), job categories (white collar, pink collar, blue collar, and none), monthly household income quartiles, and marital status. Health-related factors comprised smoking status (never, past, and current), drinking (occasionally, 2–4 times/month, and 2–4 times/week), obesity status (obese and non-obese), physical activity level (low and high, high indicating ≥ 2.5 h of moderate-intensity physical activity or ≥ 1.25 h of high-intensity physical activity per week), and history of chronic disease (stroke, cardiovascular disease including myocardial infarction and angina, hypertension, and diabetes mellitus).

### Statistical analysis

Chi-squared tests were performed to evaluate differences in the frequency and proportion of categorical variables. Multiple logistic regression analysis was conducted to examine the associations of visual and hearing acuity with LHGS, with adjustment for covariates. The results are presented as adjusted odds ratios (AORs) and 95 % confidence intervals (CIs). We examined multiplicative interaction using a series of logistic regression models with a product term and employed relative excess risk due to interaction (RERI) to investigate the presence of interactions on an additive scale of whether the combination of decreased visual and hearing acuity poses greater risk than the sum of their independent effects. RERI was calculated as the difference between the expected value based on the addition of the odds ratios of the two separate risk factors and the observed value in the dual exposed group [39], where RERI > 0 indicated a synergistic effect with decreased visual and self-reported hearing acuity. Multiple logistic regression was used to performed subgroup analyses; they were stratified by sex, age group, physical activity level, and BMI. Lastly, sensitivity analysis was performed to evaluate the association of hearing acuity considering the use of a hearing aid with LHGS (Table S1). All analyses were performed using Statistical Analysis Software (SAS, version 9.4, SAS, Inc., Cary, NC, USA), and a weighted logistic regression procedure was used to account for the complex and stratified sampling design. A two-sided *p*-value < 0.05 was considered to indicate statistical significance.

## Results

Table 1 presents the general characteristics of male and female participants with LHGS. Of the 3,075 participants, 993 (32.3 %) had LHGS. Participants with impaired visual acuity exhibited the highest proportion of LHGS (59.2 %, *n* = 87), followed by those with moderate visual acuity (48.8 %, n = 138) and those with good visual acuity (29.0 %, n = 768). The distribution of LHGS according to self-reported hearing acuity was also the same as that for visual acuity; LHGS was displayed by 137 (53.9 %) of 254 with impaired hearing acuity, 221 (40.1 %) of 551 with moderate hearing acuity, and 635 (28.0 %) of 2,270 with good hearing acuity.

**Table 1:** General characteristics of study subjects

**Variables**	**Low handgrip strength **^a^						
	**Total**		**Yes**		**No**		***p*- value**
	**N**	**%**	**N**	**%**	**N**	**%**	
	**3,075 **	**100 **	**993 **	**32.3 **	**2,082 **	**67.7 **	
**Visual acuity**							<0.001
Impaired ^b^	147	4.8	87	59.2	60	40.8	
Moderate ^c^	283	9.2	138	48.8	145	51.2	
Good ^d^	2,645	86.0	768	29.0	1,877	71.0	
**Hearing acuity (Self-reported)**							<0.001
Impaired	254	8.3	137	53.9	117	46.1	
Moderate	551	17.9	221	40.1	330	59.9	
Good	2,270	73.8	635	28.0	1,635	72.0	
**Gender**							0.722
Male	1,370	44.6	447	32.6	923	67.4	
Female	1,705	55.4	546	32.0	1,159	68.0	
**Age**							<0.001
60~64	888	28.9	128	14.4	760	85.6	
65~69	745	24.2	161	21.6	584	78.4	
70~74	622	20.2	216	34.7	406	65.3	
75~	820	26.7	488	59.5	332	40.5	
**Region**							0.006
Rural	1,648	53.6	568	34.5	1,080	65.5	
Metropolitans	1,427	46.4	425	29.8	1,002	70.2	
**Educational level**							<0.001
Under high school	2,664	86.6	901	33.8	1,763	66.2	
College or above	411	13.4	92	22.4	319	77.6	
**Job**							<0.001
White collar	190	6.2	20	10.5	170	89.5	
Pink collar	256	8.3	51	19.9	205	80.1	
Blue collar	886	28.8	252	28.4	634	71.6	
None	1,743	56.7	670	38.4	1,073	61.6	
**Household income**							<0.001
Low	1,099	35.7	494	44.9	605	55.1	
Middle low	869	28.3	252	29.0	617	71.0	
Middle high	627	20.4	145	23.1	482	76.9	
High	480	15.6	102	21.3	378	78.8	
**Marriage**							<0.001
No	834	27.1	362	43.4	472	56.6	
Yes	2,241	72.9	631	28.2	1,610	71.8	
**Smoking**							0.520
Current	333	10.8	103	30.9	230	69.1	
Past	873	28.4	272	31.2	601	68.8	
Never	1,869	60.8	618	33.1	1,251	66.9	
**Drink**							<0.001
2 ~ 4 times / week	603	19.6	182	30.2	421	69.8	
2 ~ 4 times / month	429	14.0	98	22.8	331	77.2	
Never or occasionally	2,043	66.4	713	34.9	1,330	65.1	
**Obesity Status (BMI)**^e^							<0.001
Obese (≥25)	1,152	37.5	325	28.2	827	71.8	
Normal or under-weight (<25)	1,923	62.5	668	34.7	1,255	65.3	
**Physical activity**							<0.001
Low	2,050	66.7	742	36.2	1,308	63.8	
High	1,025	33.3	251	24.5	774	75.5	
**Stroke**							<0.001
Yes	63	2.0	36	57.1	27	42.9	
No	3,012	98.0	957	31.8	2,055	68.2	
**Cardiovascular disease**^f^							0.125
Yes	196	6.4	73	37.2	123	62.8	
No	2,879	93.6	920	32.0	1,959	68.0	
**Hypertension**							<0.001
Yes	1,763	57.3	635	36.0	1,128	64.0	
No	1,312	42.7	358	27.3	954	72.7	
**Diabetes mellitus**							<0.001
Yes	703	22.9	264	37.6	439	62.4	
No	2,372	77.1	729	30.7	1,643	69.3	
**Year**							0.005
2017	1,249	40.6	368	29.5	881	70.5	
2018	1,826	59.4	625	34.2	1,201	65.8	

^a ^Defined as the 20^th^ percentile of handgrip strength of the study population (<30.4kg for male and <17.7kg for female in this study)

^b^ Best corrected visual acuity in better eye < 0.5

^c^ 0.5 ≤ Best corrected visual acuity in better eye < 0.8

^d^ Best corrected visual acuity in better eye ≥ 0.8

^e^ Obesity status defined by BMI based on 2014 Clinical Practice Guidelines for Overweight and Obesity in Korea

^f^ Defined as diagnosed diseases: myocardial infarction or angina

Abbreviations: *BMI* body mass index

Table 2 shows the results of factors associated with LHGS. Compared to participants with good visual acuity, those with moderate or impaired visual acuity demonstrated a higher risk of LHGS, after adjusting for covariates [moderate: AOR = 1.54, 95 % CI = 1.12–2.12, p = 0.009; impaired: AOR = 2.00, 95 % CI = 1.34–2.96, *p* < 0.001]. Participants with impaired hearing acuity demonstrated the highest risk of LHGS than those with good hearing acuity (AOR = 1.66, 95 % CI = 1.15–2.38, p = 0.007). The more severe the decline in sensory function, the higher the association with LHGS (p for trend in visual acuity < 0.001; p for trend in hearing acuity = 0.003). As age increased, risk of LHGS increased. Low level of physical activity was associated with an increased probability of LHGS (AOR = 1.34, 95 % CI = 1.06–1.68, *p* = 0.014), and history of stroke was associated with LHGS. However, participants with obesity were less likely to have LHGS (AOR = 0.67, 95 % CI = 0.56–0.80, p < 0.001). The sensitivity analysis for self-reported hearing acuity considering the use of hearing aid presented similar results to the main analysis (Table S1).

**Table 2:** Factors associated with low handgrip strength

Variables	Low handgrip strength ^a^				
	**AOR**	**95 % CI**			***p*-value**
**Visual acuity**					
Impaired ^b^	2.00	(1.34	–	2.96)	< 0.001
Moderate ^c^	1.54	(1.12	–	2.12)	0.009
Good ^d^	**1.00**				
* p for trend*	**< 0.001**				
**Hearing acuity (self-reported)**					
Impaired	1.66	(1.15	–	2.38)	0.007
Moderate	1.25	(1.01	–	1.55)	0.047
Good	**1.00**				
* p for trend*	**0.003**				
**Gender**					
Female	0.62	(0.45	–	0.87)	0.005
Male	**1.00**				
**Age**					
60 ~ 64	0.20	(0.14	–	0.27)	< 0.001
65 ~ 69	0.28	(0.22	–	0.37)	< 0.001
70 ~ 74	0.46	(0.35	–	0.61)	< 0.001
75~	**1.00**				
**Region**					
Rural	1.04	(0.82	–	1.33)	0.708
Metropolitans	**1.00**				
**Educational level**					
Under high school	1.03	(0.74	–	1.47)	0.845
College or above	**1.00**				
**Job**					
White collar	0.39	(0.22	–	0.69)	0.001
Pink collar	0.70	(0.48	–	1.01)	0.057
Blue collar	0.87	(0.69	–	1.10)	0.234
None	**1.00**				
**Household income**					
Low	1.38	(0.95	–	1.99)	0.091
Middle low	1.28	(0.88	–	1.87)	0.188
Middle high	1.00	(0.66	–	1.53)	0.985
High	**1.00**				
**Marriage**					
No	1.39	(1.09	–	1.76)	0.007
Yes	**1.00**				
**Smoking**					
Current	1.15	(0.80	–	1.64)	0.453
Past	0.89	(0.66	–	1.20)	0.454
Never	**1.00**				
**Drink**					
2 ~ 4 times / week	0.87	(0.66	–	1.15)	0.332
2 ~ 4 times / month	0.55	(0.39	–	0.76)	< 0.001
Never or occasionally	**1.00**				
**Obesity Status (BMI)**^**e**^					
Obese (≥ 25)	0.67	(0.56	–	0.80)	< 0.001
Normal or under-weight (< 25)	**1.00**				
**Physical activity**					
Low	1.34	(1.06	–	1.68)	0.014
High	**1.00**				
**Stroke**					
Yes	1.92	(1.01	–	3.65)	0.047
No	**1.00**				
**Cardiovascular disease**^**f**^					
Yes	0.98	(0.68	–	1.42)	0.921
No	**1.00**				
**Hypertension**					
Yes	1.05	(0.86	–	1.28)	0.637
No	**1.00**				
**Diabetes mellitus**					
Yes	1.20	(0.96	–	1.49)	0.117
No	**1.00**				
**Year**					
2017	0.71	(0.56	–	0.90)	0.005
2018	**1.00**				

AORs are adjusted for all covariates including sociodemographic, health-related factors, history of chronic disease, and year of survey.

^a^ Defined as the 20th percentile of handgrip strength of the study population (< 30.4 kg for male and < 17.7 kg for female in this study)

^b^ Best corrected visual acuity in better eye < 0.5

^c^ 0.5 ≤ Best corrected visual acuity in better eye < 0.8

^d^ Best corrected visual acuity in better eye ≥ 0.8

^e^ Obesity status defined by BMI based on 2014 Clinical Practice Guidelines for Overweight and Obesity in Korea

^f^ Defined as diagnosed diseases: myocardial infarction or angina

Abbreviations: *AOR* adjusted odds ratio; *CI* confidence interval; *BMI* body mass index

Table 3 describes the synergistic interaction of visual and hearing acuity with LHGS on an additive scale. Despite no multiplicative interaction, we observed a statistically significant additive interaction between visual and hearing acuity on LHGS. In the analyses with good visual and hearing acuity as the reference category, impaired visual or hearing acuity alone were associated with increased odds of LHGS (impaired visual acuity alone: AOR = 1.72, 95 % CI = 1.06–2.78, *p* = 0.027; impaired hearing acuity alone: AOR = 1.56, 95 % CI = 1.04–2.36, *p* = 0.034). However, for participants with both risk factors, the AOR of LHGS increased significantly to 8.38 (95 % CI = 2.12–33.20, *p* = 0.003). The RERI was 2.61 (95 % CI = 2.52–2.70, *p* < 0.001). In other words, when impaired visual and hearing acuity were present together, the odds ratio was greater than the sum of the individual effects.

**Table 3:** Interactions between visual and hearing acuity in relation to low handgrip strength

Variables	Low handgrip strength		
	**Hearing acuity**		
	Good	Moderate	Impaired
	AOR (95 % CI)	AOR (95 % CI)	AOR (95 % CI)
**Visual acuity**			
Good	1.00 (reference)	1.22 (0.95–1.58)	1.56 (1.04–2.36)^*^
Moderate	1.51 (1.02–2.23)^*^	1.95 (1.14–3.34)^*^	2.50 (1.22–5.10)^*^
Impaired	1.72 (1.06–2.78)^*^	2.77 (1.22–6.26)^*^	8.38 (2.12–33.20)^**^

AORs are adjusted for all covariates including sociodemographic, health-related factors, history of chronic disease, and year of survey.

Measure of interaction on additive scale: RERI = 2.61 (95 % CI: 2.52–2.70); *p* < 0.001

Significance of multiplicative interaction: *p* < 0.129

^*^p-value < 0.05; ^**^p-value < 0.01

Abbreviations: *AOR* adjusted odds ratio; *CI* confidence interval

**Fig. 2:**
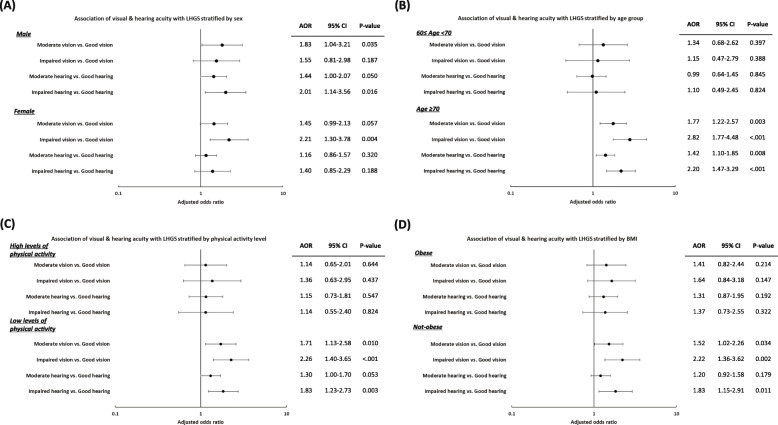
Subgroup analyses stratified by sex, age group, physical activity level, and BMI. LHGS, low handgrip strength; BMI, body mass index; AOR, adjusted odds ratio; CI, confidence interval

Figure 2 presents the results of the stratified analyses. LHGS reported prominent associations with hearing acuity among male and with visual acuity among female participants. Moreover, older adults reported a prominent association between sensory dysfunction and LHGS. Individuals who were not obese or had low levels of physical activity in the moderate and impaired visual acuity group showed significantly increased odds of having LHGS (not obese: moderate visual acuity, AOR = 1.52, 95 % CI = 1.02–2.26, *p* = 0.034; impaired, AOR = 2.22, 95 % CI = 1.36–3.62, *p* = 0.002; low level of physical activity: moderate visual acuity, AOR = 1.71, 95 % CI = 1.13–2.58, p = 0.010; impaired, AOR = 2.26, 95 % CI = 1.40–3.65, *p* < 0.001).

## Discussion

This representative population-based study provided evidence that older Koreans with impaired visual acuity and those with moderate visual acuity had a higher risk of reduced muscle strength, as indicated by LHGS. Moreover, impaired and moderate self-reported hearing acuity were significantly associated with increased risk of LHGS, compared to good hearing acuity. The association between sensory organ function and LHGS demonstrated a progressively increasing pattern as visual and hearing acuity deteriorated. Our results are similar to those of previous research that poor self-rated eyesight was associated with LHGS, and multiple sensory impairments, including hearing impairment, were associated with lower adjusted mean handgrip strength [23, 24]. However, prior studies only divided visual and hearing acuity in a dichotomous manner to evaluate the association with handgrip strength, and there was no study in which hearing impairment alone was related to LHGS.

Notably, a combined interaction of impaired visual and hearing acuity that was greater than the sum of their individual impacts was identified. Compared to participants with good visual and hearing acuity, those with concomitant impaired visual and hearing acuity had up to eight times greater odds of having LHGS. Several studies have shown that combined or concurrent impaired visual and hearing acuity are associated with cognitive and functional decline [39], and functional dependence [40]. However, as far as we know, there have been no studies that have confirmed the synergistic association with LHGS by distinguishing the degree of visual and hearing acuity. We further analyzed the additive interaction to evaluate the synergistic association of visual and hearing acuity with handgrip strength using RERI; there was a strong additive interaction between impaired visual and hearing acuity. Since additive interaction depicts a higher absolute excess of cases than multiplicative interaction, it is very important to estimate the additive scale from a biological and public health perspective [41]. Thus, older people who have both visual and hearing impairments may need faster and more appropriate intervention to prevent deterioration of physical function.

The mechanism of the association between decreased visual and/or hearing acuity and LHGS remains unclear. There are several hypotheses proposing that decreased visual and/or hearing acuity are related to LHGS or overall muscle strength. Visual acuity plays a leading role in balance control by providing the nervous system with continuously updated information about the position and movement of body segments in relation to each other and the environment [42]. Consequently, older people with decreased visual acuity tend to avoid physical activity because of the fear of falling, and avoidance of physical activity causes decreased muscle strength as assessed by handgrip strength [43, 44]. In the case of hearing, it is possible that sensory deprivation, changes in resource allocation, and social isolation owing to hearing impairment may have affected LHGS [45]. However, one of the most persuasive mechanisms is that decreased visual and/or hearing acuity are early physiologic markers of LHGS. This could be explained using the common-cause theory, which states that one or more factors contribute to the development of both sensory impairments and LHGS. Aging might be one such factor [23, 25]. Vascular disease or inflammation could be another potential common causative factor [46–48]. Furthermore, sensory and muscle strength may share more than aging and vascular inflammation or disease as common causes. Finally, sensory impairments might be an early sign or a surrogate marker of the development of frailty or the aging process, which can be measured by handgrip strength [49, 50].

 The subgroup analyses demonstrated prominent associations of LHGS with hearing acuity in male participants and with visual acuity in female participants. It is difficult to explain these sex-specific associations of visual and hearing acuity with LHGS. Sex-differences have been found in the predictors of LHGS [51], and these differences need to be studied further to examine the potential underpinnings of sex-specific associations of sensory impairment with physical fuction. Meanwhile, participants aged > 70 years reported a more pronounced association between sensory organ dysfunction and LHGS. This may be the effect of age-related changes in cross-modal deactivations [52], but further research is needed. The association of decreased visual and hearing acuity with LHGS was greater in participants with low levels of physical activity. Low physical activity level is a well-known factor predicting decreased overall muscle strength [53], and this result indicated that decline of sensory function and low physical activity level are more relevant to LHGS than low physical activity level only.

Several methods can be employed to determine cut-off values for LHGS, which reflect muscle strength as part of the defining criteria for sarcopenia [32, 54–56], and some studies have reported reference values for handgrip strength in the Korean population [57, 58]. In our study, the criteria for LHGS were based on the 20th percentile of the study population [32]. Thus, LHGS was defined as < 30.4 kg for male and < 17.7 kg for female. We did not use the reference values because the data from KNHANES are acquired annually and factors associated with handgrip strength, such as nutritional or smoking status, change with the passage of time. Hence, it was determined that the 20th percentile of the study population was a better representative of the total population at the time of the study, rather than the reference values determined in a particular year.

This study has the following limitations. First, the cross-sectional design of this study cannot confirm the causality between sensory function decline and LHGS. However, we mitigated these limitations by employing appropriate methods including adjusting sociodemographic characteristics and well-established risk factors for LHGS and evaluating dose-response relationship. Second, handgrip strength estimation may vary depending on the position or method of handgrip measurement [59]. Third, participants only underwent automated refraction using an auto-refractor/keratometer and thus, visual acuity might not have been fully corrected. Fourth, this study employed self-reported measures for evaluating hearing acuity, which may have incurred response bias resulting in validity issues. Popularly, hearing impairment is defined as pure-tone average thresholds > 40 dB using pure-tone audiometry [60]. However, pure-tone audiometry was not used in the 2017 and 2018 KNHANES. Several existing studies have employed similar self-reported measures to categorize hearing groups [36]; however, we tried to enhance the validity using sensitivity analysis. Additional longitudinal studies are needed to confirm the effect of sensory impairments on physical function such as handgrip strength through a method that can objectively evaluate sensory organ function. Fifth, we could not confirm the associations of LHGS with cognitive impairment in Koreans [61], as KNHANES did not contain information on cognitive function. However, KNHANES participants underwent physical examinations and answered a lengthy questionnaire; thus, it might be difficult for individuals with reduced cognitive function to participate. Lastly, although gait impairment is considerably affected by sensory organ dysfunction, KNHANES lacks such information, and it could not be evaluated.

Nevertheless, the present study has several strengths. First, to the best of our knowledge, this is the first study to identify the synergistic association of visual and hearing acuity with LHGS in the older Korean population. Furthermore, our study is based on a nationally representative survey and random cluster sampling. This makes the data more reliable and representative of the Korean population. Additionally, our findings indicate that awareness is needed regarding risk of reduced muscle strength in individuals with decline of sensory function, even if the level of sensory function decline is minor and not severe. The underlying mechanism and directionality in this relationship needs to be confirmed in future biological or longitudinal studies. Furthermore, if sensory function decline is found in the older participants in a clinical setting, it may be helpful to develop a program such as exercise prescription to prevent muscle weakness in the future.

## Conclusions

The current study found that older people with decreased visual or hearing acuity had a higher risk of having LHGS than those with good visual or hearing acuity, and the associations differed based on the level of sensory function decline. Additionally, our results provided further evidence that concomitant decreased visual and hearing acuity may be synergistically associated with LHGS, which is considered an estimate of overall muscle strength. This implies that biological or longitudinal prospective studies are needed to understand the effect of decline of sensory organ function on reduced muscle strength. Moreover, in older people clinically diagnosed with sensory function decline, it may be necessary to develop a program to prevent future muscle weakness.

## Supplementary Information

**Additional file 1.**

## Data Availability

All the KNHANES data used this study are available to the public and can be seen in the KNHANES official website (https://knhanes.kdca.go.kr/knhanes/main.do).

## Abbreviations

LHGS: Low handgrip strength
ADL: Activities of daily living
BMI: Body mass index
KNHANES: Korea National Health and Nutrition Examination Survey
AORs: Adjusted odds ratios
CIs: Confidence intervals
RERI: Relative excess risk due to interaction
