# Supplementary material for: Synergistic associations of visual and self-reported hearing acuity with low handgrip strength in older adults: a population-based cross-sectional study
Source: BMC Geriatr. 2021 Sep 25;21:513. doi: 10.1186/s12877-021-02470-w (PMC8466740; doi:10.1186/s12877-021-02470-w)
Supplement: Supplementary file 1 — Additional file 1. [file 12877_2021_2470_MOESM1_ESM.docx]

| **Table S1. Sensitivity analysis of association of hearing acuity considering the use of hearing aids with LHGS** | | | | | | | | | |
| --- | --- | --- | --- | --- | --- | --- | --- | --- | --- |
| **Variables** | **Low handgrip strength ^a^** | | | | | | | | |
|  | **N** | **%** | **AOR** |  | **95% CI** | | |  | **P-value** |
| **Visual acuity** |  |  |  |  |  |  |  |  |  |
| Impaired ^b^ | 87 | (59.2) | 1.99 |  | (1.34 | – | 2.98) |  | <0.001 |
| Moderate ^c^ | 138 | (48.8) | 1.54 |  | (1.12 | – | 2.12) |  | 0.009 |
| Good ^d^ | 768 | (29.0) | **1.00** |  |  |  |  |  |  |
| *p for trend* |  |  | **<0.001** |  |  |  |  |  |  |
| **Hearing acuity** |  |  |  |  |  |  |  |  |  |
| Impaired ^e^ | 146 | (52.9) | 1.62 |  | (1.15 | – | 2.27) |  | 0.006 |
| Moderate ^f^ | 212 | (40.1) | 1.25 |  | (1.00 | – | 1.56) |  | 0.048 |
| Good ^g^ | 635 | (28.0) | **1.00** |  |  |  |  |  |  |
| *p for trend* |  |  | **0.003** |  |  |  |  |  |  |
| AORs are adjusted for all covariates including sociodemographic, health-related factors, history of chronic disease, and year of survey. | | | | | | | | | |
| ^a^ Defined as the 20th percentile of handgrip strength of the study population (<30.4kg for male and <17.7kg for female in this study). | | | | | | | |  |  |
| ^b^ Best corrected visual acuity in better eye < 0.5 | | | | | | | |  |  |
| ^c^ 0.5 ≤ Best corrected visual acuity in better eye < 0.8 | | | | | | | |  |  |
| ^d^ Best corrected visual acuity in better eye ≥ 0.8 | | | | | | | |  |  |
| ^e^ Participants with moderate or considerable trouble with hearing aid and almost deaf  ^f^ Participants who felt a little uncomfortable with their subjective hearing but did not use a hearing aid  ^g^ Participants with good or normal hearing without hearing aid | | | | | | | | | |
| Abbreviations: LHGS, low handgrip strength; AOR, adjusted odds ratio; CI, confidence interval; BMI, body mass index | | | | | | | | | |
